# Initial ecological change in plant and arthropod community composition after wildfires in designated areas of upland peatlands

**DOI:** 10.1002/ece3.9771

**Published:** 2023-02-11

**Authors:** Ruth Kelly, W. Ian Montgomery, Neil Reid

**Affiliations:** ^1^ Institute for Global Food Security (IGFS) School of Biological Sciences Queen's University Belfast Belfast UK; ^2^ Environment and Marine Sciences Division Agri‐Food and Biosciences Institute (AFBI) Belfast UK

**Keywords:** beetles, blanket bog, burning, dry heath, moorland, recovery, spiders, vegetation, wet heath, wildfire

## Abstract

Wildfires are an increasing concern due to rising temperatures and incidence of droughts associated with changing climate, poor land management, and direct human interference. Most studies of the impact of fire on temperate heathland and bog examined the consequences of controlled or prescribed burning. Less is known about the impacts of uncontrolled wildfires on sites designated for their conservation value. We examined the initial impact and short‐term trajectory (3.5 years) of cool temperate peatland plant and arthropod communities on designated upland sites in Northern Ireland following wildfires, that is, unplanned with respect to where and when they occur, severity, and duration. These near simultaneous wildfires were often due to a failure to control prescribed burns. Wildfires were associated with a loss of blanket bog and heath indicator species. Broad vegetation groups showed initial recovery characterized by a decrease in bare ground and increasing cover of shrub species and bryophytes. However, at a species level, *Sphagnum* spp and bryophyte communities, which are central to peatland ecosystem functioning, showed no sign of recovery to prefire composition. Rather, bryophyte communities became more divergent over the course of the study and were mainly characterized by increased abundance of the alien pioneer acrocarp *Campylopus introflexus*. Similarly, composition of arthropod communities (ground beetles and spiders) differed between burnt and unburnt areas and showed no evidence of a return to species composition in unburnt areas. The nationally rare beetle *Carabus nitens* was more common in the aftermath of wildfire. *Synthesis*. Whilst, long‐term recovery was not investigated, these short‐term changes suggest enduring detrimental impacts on the distinctive communities associated with peatlands, primarily through the loss of *Sphagnum* spp*.*, affecting ecosystem services such as carbon sequestration and water and soil retention. It may not be possible to restore exact prefire species composition of plant and animal communities. We suggest a precautionary approach involving management of upland vegetation, public education, and vigilance, to prevent further wildfires and protect these key upland habitats.

## Graphical abstract

Wildfires affect richness and abundance in moorland plant and arthropod communities. Critically, peat‐forming, Sphagnum communities, showed no sign of recovering pre‐fire composition.

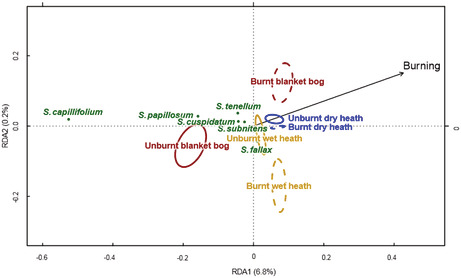

## 1 INTRODUCTION

The impact of wildfires on temperate and boreal peatlands is of increasing concern from scientific and conservation perspectives (Chuvieco et al., 2014; Moritz et al., 2014; Turetsky et al., 2015). Increased temperatures and drought due to climate change increase the risk of wildfires in temperate regions and alter the capacity of natural habitats to cope with such events (Fernandez‐Anez et al., 2021; Krawchuk et al., 2009). Drained and drying boreal peatland subject to wildfires release more carbon into the atmosphere with global consequences (Harris et al., 2020; Lin et al., 2021). Whilst most severe impacts may occur in higher latitudes and hotter, drier climates, increased temperature and decreased precipitation in summer months in temperate countries (Blenkinsop & Fowler, 2007; Murphy & Fealy, 2010) may also result in significantly more, higher intensity wildfires (Albertson et al., 2011; Arnell et al., 2021). European wildfires are predominantly anthropogenic in origin with more than 95% of wildfires started by people (Birot, 2009; McMorrow et al., 2009). Thus, wildfires represent an interaction between people, landscape, and climate, which can be mitigated by management.

Research is needed on impact and potential restoration of post‐wildfire ecosystems. There is a particular dearth of studies on animals (Driessen & Kirkpatrick, 2017) and plant communities, to bridge the interests of local management and global concerns (Jefferson et al., 2020; Phelps et al., 2021). Some wildfires play a vital role in determining the distribution of fire‐tolerant species and contributing naturally to atmospheric and terrestrial carbon budgets (Flanagan et al., 2020). Upland heather moorland, characterized by low growing vegetation on acidic soils, is of international conservation importance in the British Isles (Joosten, Szallies, & Tegetmeyer, 2016; Usher & Thompson, 1993), containing 13 vegetative communities (EC Habitats Directive EEC, 1992), with six communities occurring only in Great Britain and Ireland (Thompson et al., 1995). These plant communities provide important habitats for fauna, including a high diversity of invertebrate species (Usher, 1992), mammal species including the pygmy shrew (*Sorex minutus*) and mountain hare (*Lepus timidus*), and bird species. Skylarks (*Alauda arvensis*) and Meadow Pipits (*Anthus pratensis*), which can occur at high densities in upland heather, along with eight other species including Red Grouse (*Lagopus lagopus scoticus*), Hen Harrier (*Circus cyaneus*), and Golden Plover (*Pluvialis apricaria*), are protected under the EC Birds Directive (EEC, 1979). Peatlands of northwest Europe are also of cultural and historical significance (Odgaard, 1994; Pyne, 1997) and have a significant role as carbon sinks (Joosten, Sirin, et al., 2016).

Palaeoecological studies based on plant remains and charcoal in peat up to 7000 years ago suggest that ombrotrophic peatland is generally resistant to fire (Magnan et al., 2012), affecting bryophyte and vascular plant succession only over decades or several hundreds of years after a fire (Gałka et al., 2022; Kuhry, 1994; Sillasoo et al., 2011). Previous UK studies on the impacts of fire focused mainly on prescribed burning on heathland sites with a long history of human influence in the form of grazing by livestock, and burning and cutting vegetation (e.g., Davies et al., 2010; Harris et al., 2011; Tharme et al., 2001). There is little information on the impact of wildfires, on extensively managed sites, particularly blanket bog. Furthermore, earlier research focused mainly on vascular plant species with less information on the impacts of burning on bryophyte species, which are critically important in the formation of peat, and invertebrate fauna which characterize upland habitats.

Prescribed burning aims to improve upland grazing for livestock and red deer (*Cervus elaphus)*, or for the management of Red Grouse (*Lagopus lagopus scoticus)*. Degenerate stage heather (*Calluna vulgaris* and *Erica* spp.) is burnt swiftly at low intensity ensuring fire does not penetrate the peat (Davies et al., 2010; Scottish Natural Heritage, 2017; Whitehead et al., 2021). Prescribed burning can optimize regrowth of *C. vulgaris* with respect to season, autumn, and age between 6 and 10 years (Miller & Miles, 1970). This creates a mosaic comprised of different successional stages of burnt heather, interspersed with unburnt heather. Prescribed burning created and maintained heathlands over thousands of years. However, it increases landscape and species diversity benefiting populations of some species but not others (Langholm Moor Demonstration Project Board, 2019; Ludwig et al., 2019). For example, *Sphagnum* spp. are more abundant in areas burnt 8–10 years earlier in the North York Moors of England (Whitehead et al., 2021), numbers of Red Grouse are enhanced by controlled, rotational burning (Robertson et al., 2017) but abundance of Meadow Pipit (*Anthus pratensis*) decreases (Smith et al., 2001). Wildfires, which are often out‐of‐control, prescribed burning, may involve uncontrolled, expansive, and prolonged burning, penetrating peat and changing physical and chemical structure of soil and plant and animal communities (Kelly et al., 2018; Maltby et al., 1990). Thus, low‐diversity landscapes perhaps comprising only one vegetational successional stage are created. Wildfires can also reduce seedbank species richness with specialist wetland species initially failing to recover (Kelly et al., 2016).

This study examines the effects of wildfire on upland moorland plant and arthropod communities over three‐and‐a‐half years in burnt areas compared with adjacent unburnt areas within sites of conservation concern. This is an opportunistic study to determine whether vegetation and invertebrate communities start to recover rapidly after wildfires. The broad objective was to survey changes in plant and arthropod community composition after wildfires, in designated upland sites of conservation interest, in relation to habitat structure especially vegetation height, soil conditions, and management. We hypothesized that: (1) wildfires immediately alter plant and arthropod communities with incomplete recovery in the short term; (2) heathland and bogland specialist species such as *Sphagnum* spp. are more affected by wildfires than generalist plant species; and (3) community composition of major arthropod taxa changes such that abundance of some species decreases whilst others may increase in burnt areas.

## 2 METHODS

### 2.1 Site and quadrat selection

GIS mapping was used to identify six Areas of Special Scientific Interest (ASSIs) within which large wildfires occurred in early summer 2011 and contained areas of both upland heath and blanket bog habitats (Table 1). The location and size of these fires was derived from satellite data using the European Forest Fire Information System (EFFIS, http://effis.jrc.ec.europa.eu Table 1). Prefire condition assessments, conducted by the Northern Ireland Environment Agency (NIEA), Department of Agriculture, Environment and Rural Affairs (DAERA), were used to classify quadrats into three EU Annex I habitat classes using the Joint Nature Conservation Committee National Vegetation Classification (NVC) system for UK habitats (Averis, 2004; JNCC, 2009). Annex I habitats included were “blanket bog” (*n* = 72), “Northern Atlantic wet heaths with *Erica tetralix*” (*n* = 24), and “European dry heaths” (*n* = 20). In the study region, these habitats have some dominant species in common (e.g., *Calluna vulgaris, Eriophorum angustifolium, and Sphagnum capillifolium*) and are distinguished based on a combination of soil, vegetation, and hydrographic characteristics. Blanket bog is primarily distinguished from wet and dry heath by the depth of peat (>50 cm). Where it is not possible to determine peat depth, a higher frequency of *Sphagnum* moss species and hare's tail cotton grass (*Eriophorum vaginatum*) are used to distinguish blanket bog from heath. Wet and dry heath are distinguished from one another based on hydrological characteristics and associated plant communities. Wet heath communities are characterized by mixtures of heather species (*Calluna vulgaris, Erica tetralix*) and graminoids such as *Trichophorum cespitosum* and *Molinia caerulea,* over an understory of pleurocarpic and *Sphagnum* moss species. Dry heath is typically dominated by a range of dwarf shrubs such as Ling heather (*Calluna vulgaris*), bell heather (*Erica ciner*ea), bilberry (*Vaccinium myrtillus*), and occasionally Crowberry (*Empetrum nigrum*) or western gorse (*Ulex gallii*), whilst it may contain *Sphagnum* moss species, these are generally less abundant or diverse than in wet heath or blanket bog habitats. A total of 116 quadrats (2 × 2 m) were included in the study selected randomly from the quadrats, which had been previously surveyed by the Northern Ireland Environment Agency. Sixty‐seven quadrats were in areas burnt during 2011 and forty‐nine in nearby unburnt areas, such that burnt and unburnt areas were sampled within each site. Two additional quadrats were surveyed at Cuilcagh in 2013 to replace two previously unburnt quadrats at that site which were burnt in April 2013. The number of quadrats within each habitat was determined in proportion to the occurrence of that habitat at each site. The mean distance between quadrats within sites was 1.77 km (min = 0.07 km, max = 6.07 km).

**TABLE 1:** Areas of Special Scientific Interest (ASSIs) on which wildfires occurred in the spring or early summer of 2011; showing area of fire and percentage damaged

ASSI	County	Area burnt (km^2^)	%of ASSI burnt	Designated habitat feature	Species feature
Cuilcagh Mountain	Fermanagh	1.44	5	Blanket bog, Dry heath, Inland rock, Montane heath, Dystrophic lakes, Wet heath	Golden Plover, Higher plant assemblage, Invertebrate assemblage
Eastern Mournes	Down	9.31	12	Blanket bog, Dry heath, Inland rock, Montane heath, Oligotrophic lakes, Wet heath	Fungi assemblage, Higher plant assemblage, Invertebrate assemblage
Glennasheevar	Fermanagh	1.72	63	Blanket bog, Wet heath	Marsh Fritillary, Invertebrate assemblage
Mullaghcarn	Tyrone	11.20	54	Blanket bog, Wet heath, Dry heath, Oakwood, Dystrophic lakes	
Slieve Beagh	Fermanagh & Tyrone	11.67	61	Blanket bog, Dry heath, Dystrophic lakes	Invertebrate assemblage
Slieveanorra/Croaghan	Antrim	1.03	6	Blanket bog, Wet heath, Dry heath	Merlin, Hen Harrier
TOTAL/MEAN		38.01	32		

*Note*: Habitat and species selection features are also listed. Burnt area data were extracted from the European Forest Fire Information System (EFFIS). For further details and site maps, see https://www.daera‐ni.gov.uk/protected‐areas/. A map of sites is also in a related paper (Kelly et al., 2016).

### 2.2 Field survey

Vegetation quadrats were surveyed between June and October in 2012, 2013, and 2014, corresponding to approximately one, two, and 3 years after wildfires. Species‐level plant data for vascular plants and bryophytes were recorded as percentage cover within each quadrat and *Cladonia* lichens were recorded at genus level.

In 2013 and 2014, invertebrate pitfall trapping was conducted in all six ASSIs (Table 1). Twenty four pitfall traps were placed level with the soil surface. Traps were placed in four transects with three traps in each, at two locations within burnt and unburnt areas at each site. Trapping began in late May and ended in early October with traps in‐situ for 4 weeks, representative of the summer activity months (June, July, August, and September). At Slieveanorra, trapping began in July due to lack of access permissions during the early part of the bird‐nesting season. Polyethylene containers (15 cm deep with 10 cm opening) were primed with 5 cm of nontoxic antifreeze (approximately 1:1 ratio of monopropylene glycol, and water). Each container was covered with 1 cm plastic mesh to exclude small mammals and a 15 cm square of corrugated plastic sheet to exclude excess rainwater. Due to repeated human disturbance of the traps at the Eastern Mournes site, this site was excluded from the final analysis. For similar reasons, individual traps from Glennasheevar and Slievebeagh could not be included in June 2013, and there was no sampling at Slievanorra in June due to access restrictions to sampling. Ground beetles (Carabidae) were identified to species level for the survey months June, July, August, and September of both years (*n* = 396). As spider identification to species level requires a greater degree of technical skill and expert verification, it was necessary to reduce the number of months for which spiders were identified. Therefore, spider species were identified for the survey months June and September of both years to represent the beginning and end of the summer season (*n* = 212 traps).

### 2.3 Statistical analyses

#### 2.3.1 Plant functional groups

Changes in composition of plant functional groups (Harrison et al., 2010) in terms of percentage cover before and after burning were analyzed by partial Redundancy Analysis (pRDA, Legendre & Legendre, 2012). These groups were as follows: shrubs, bracken, herbs, graminoids, *Sphagnum*, other bryophytes, and *Cladonia* lichens. Cover measurements from prewildfire NIEA condition assessments (Glennasheevar 2005, Cuilcagh 2006, Slieveanorra and Mullaghcarn 2007, Eastern Mournes and Slievebeagh 2008) and all three survey years (2012, 2013, and 2014) were included. Vegetation matrices were transformed using a Hellinger transformation to meet the assumptions of pRDA. Explanatory factors and variables were as follows: fixed factors of Burning (Burnt or Unburnt) and Habitat (blanket bog, dry heath, or wet heath) and covariates of Year, Altitude (m), Slope (° from horizontal), Solar heat load Index, and Grazing. The interactions between Burning and Grazing, Burning and Habitat and Burning and Slope, were also fitted to account for potentially different effects of wildfires in difference contexts (i.e., on more or less grazed sites, different habitats, or steeper slopes). The interaction between Burning and Year was also fitted to account for differences in community change over time between burnt and unburnt quadrats. Topographical variables (i.e., Altitude and Slope) were extracted from GIS raster files at a 25 m resolution. Heat Load was a measure of local temperature resulting from solar radiation, slope, and aspect specifically designed for use in vegetation science (McCune & Dylan, 2002). Here, the Heat Load Index was calculated according to Equation 3 in McCune and Dylan (2002) based on raster data at a 25 m resolution. This equation was chosen as it was most suitable for areas with slopes of less than 60° and latitudes of between 30 and 60°. Whilst this metric does not account for small‐scale variation in temperature caused by, for example, local shading or surface reflectance, it provides a useful measure of differences in thermal environments at a landscape scale (e.g., between north and south facing, or shallow and steep slopes). Grazing intensity levels were assessed at each quadrat based on the quantity of dung present in each quadrat (Campbell et al., 2004; Davis & Coulson, 2016; Welch et al., 2006) and were ranked on a three‐level ordinal factor scale of None <Low <High. Specifically, None/very low = 0 droppings, Low = 1–10 droppings, High >10 droppings. All explanatory variables were rescaled to units of standard deviation prior to model fitting. Site was fitted as a “conditional” variable in the pRDA, meaning that differences between sites were accounted for by partial ordination, prior to fitting all other explanatory factors and variables. The most parsimonious pRDA model was selected based on model AIC values using a forward stepwise selection procedure. Additional explanatory variables were added to the null model where they significantly improved the model fit based on permutation testing (inclusion criteria *p* < .05). Final variables in pRDA models were tested against a null hypothesis that communities did not differ from randomly assembled communities within sites, using permutation based testing with “site” as a strata (*n* = 999 permutations). Thus, *p*‐values <.05 indicate that variables in the model explain more variation than expected if species were randomly assigned to communities within each site.

#### 2.3.2 Plant species richness and community composition

Species‐level analysis of the vegetation community was conducted on three different subsets of the data, each corresponding to a different part of the vegetation community. These three groups were as follows: vascular plants, bryophytes other than *Sphagnum* spp. mosses (hereafter, “non‐*Sphagnum*” bryophytes), and *Sphagnum* spp. For each of these plant groups, differences in species richness were assessed using Generalized Linear Mixed Models (GLMM; Stroup, 2012) and differences in community composition were assessed using pRDA. Generalized Linear Mixed Models were initially fitted using a Gaussian response distribution. Where model residuals were not normally distributed (based on a Shapiro–Wilk test), full models were refitted by Laplace estimation with Poisson, negative binomial, zero inflated Poisson, and zero inflated negative binomial response distributions, and the optimal response distribution was chosen based on the lowest model Akaike information criterion (AIC) value. Subsequently, all possible subsets of the explanatory variables in the global model were fitted and compared based on AIC. Model averaging was used to estimate the relative importance and estimated effect size of predictor variables from models within 2 ΔAICc of the top model. Effects sizes of averaged models were estimated using the zero method of model averaging meaning that effects sizes were averaged across all models with a value of zero in models in which they do not occur. This provides a conservative estimate of the effect sizes of variables, which occur in only a small proportion of models in the top model set (Anderson & Burnham, 2002). For species community analyses, explanatory factors and variables and pRDA model selection procedure were the same as those above for functional plant groups, except years prior to the wildfire, were not included as the preburn condition assessments did not provide species‐level identification of all taxa.

#### 2.3.3 Arthropod abundance, richness, and community composition

Two key groups of predatory invertebrates were analyzed, the ground beetles (Carabidae) and spiders (Araneae), for five sites 2 and 3 years after wildfires. Differences in abundance and richness of ground beetle and spider species were assessed using a GLMM approach. Differences in community composition were assessed using pRDA. Explanatory variables in GLMM's and pRDA were Burning (Burnt/Unburnt), Year (2013/2014), and Month (June/July/August/September for ground beetles and June/September for spiders). Interactions between Burning and Month and between Burning and Year were also fitted to account for potentially differing impacts in different seasons or differences in burnt areas between years (e.g., recovery or increasing divergence). In GLMMs, Transect was fitted as a random factor nested within Site to account for similarity between samples within transects and sites. In pRDA analysis, Site was fitted as a “conditional” factor where differences between sites were accounted prior to fitting all other explanatory factors. Further explanatory variables relating to topography, heat load, and grazing used in plant models above were not used in the arthropod models, because of the lack of variation in these variables between the trapping transects within sites. Model selection procedures for both GLMM and pRDA models were as above for plant species analysis.

Further details of statistical methods are presented by Kelly et al. (2016). All analyses were conducted using R 3.1.1 (R Core Team, 2020). Generalized Linear Mixed Models were fitted using the R packages glmmADMB (v 0.7.7) and MuMIn (v1.9.11). pRDA were calculated in the R package vegan(v2.0–9). Datasets are available at: http://dryad.org/10.5061/dryad.fbg79cnzz.

## 3 RESULTS

### 3.1 Functional plant groups

**FIGURE 1:**
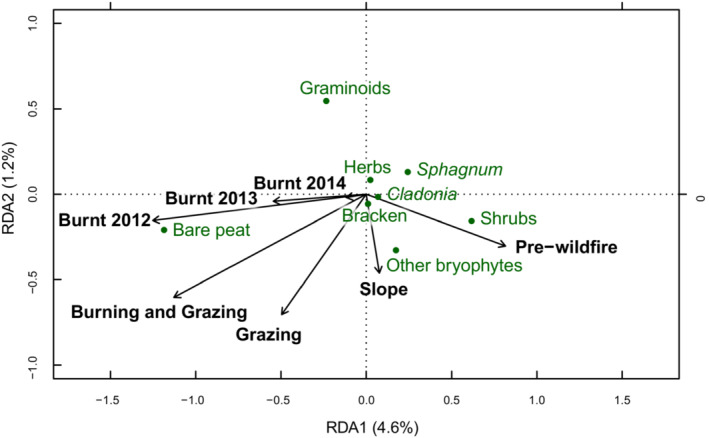
Partial Redundancy Analysis (pRDA) biplot showing relationship between burning and grazing and the cover of functional plant groups, when site and quadrat were accounted for by pRDA.

Variation in cover of plant functional groups (Figure 1) was best explained by Burning (*p* = .001), Grazing (*p* = .001), Habitat (*p* = .001), Slope (*p* = .006), and Year (i.e., preburn/2012/2013/2014) (*p* = .001). There was a significant interaction between Year and Burning (*p* = .001) and between Burning and Grazing (*p* = .001). These interactions indicate that the rate of change in functional plant groups differed between Burnt and Unburnt quadrats (burnt areas changed over time, whilst unburnt areas remained similar) and that grazing pressure affected burnt and unburnt areas differently. There was also an interaction between Habitat and Year, indicating that the rate of change in functional plant groups differed between habitat types (*p* = .004). The full model explained 25.2% of the variation in functional plant groups, after accounting for differences between sites. Burning and its interactions with Grazing and Year explained a total of 12.5% of the variation in functional plant groups.

**FIGURE 2:**
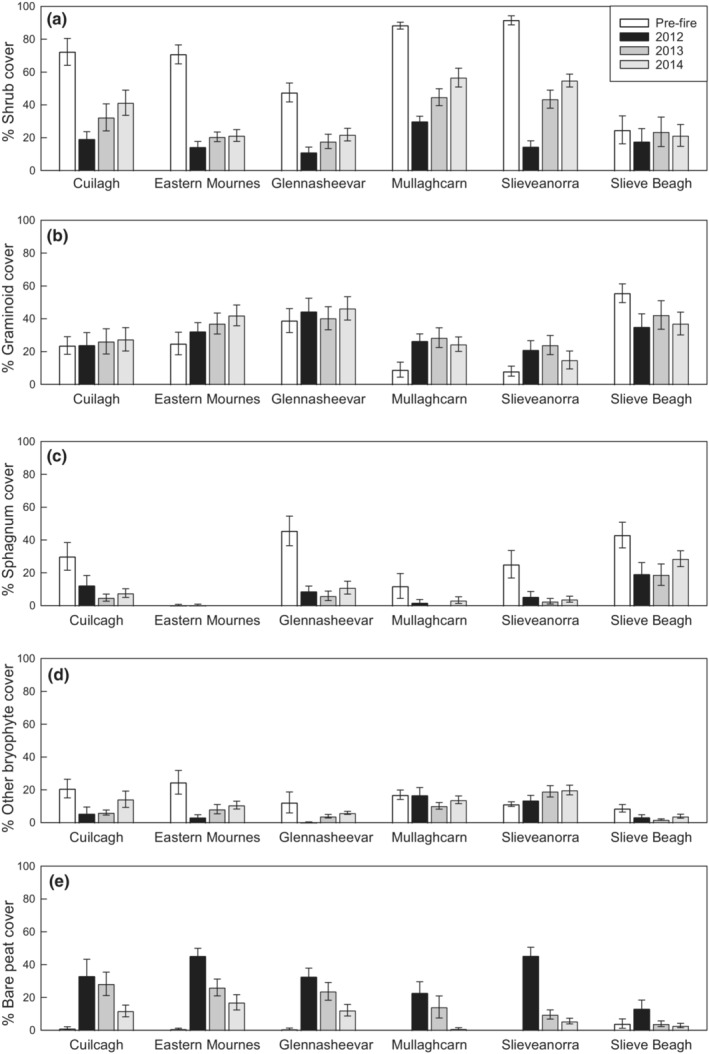
Recovery of vegetation cover types at each ASSI; (a) shrub cover, (b), graminoid cover, (c) *Sphagnum* cover, (d) bryophyte cover, and (e) bare soil. White bar shows mean pre‐survey cover in areas which were subsequently burnt, black bars show mean of burnt quadrats in 2012, mid‐gray bars show mean of burnt quadrats in 2013, light gray bars show mean of burnt quadrats in 2014. Error bars show standard error. *Cladonia* and bracken (*Pteridium aquilinum*) not shown, accounted for a mean cover of <2% and <4% respectively, at all sites.

Prewildfire and unburnt quadrats had a higher cover of shrub species, bryophytes (including *Sphagnum*), and *Cladonia* lichens than burnt quadrats (Figures 1 and 2). Burning was associated with higher cover of bare peat and a slight increase in graminoid cover. Initial recovery of plant functional groups took place in burnt areas from 2012 to 2014. This was characterized by increasing cover of shrubs and bryophytes including *Sphagnum* spp. Grazing was primarily associated with a reduction in graminoid and *Sphagnum* cover and an increase in bare peat, but also resulted in a higher cover of non‐*Sphagnum* bryophytes. When the impacts of grazing and burning occurred together, graminoid cover was lower than following burning alone. Recovery rates also differed between ASSI's (Figure 2).

### 3.2 Vascular plants

**FIGURE 3:**
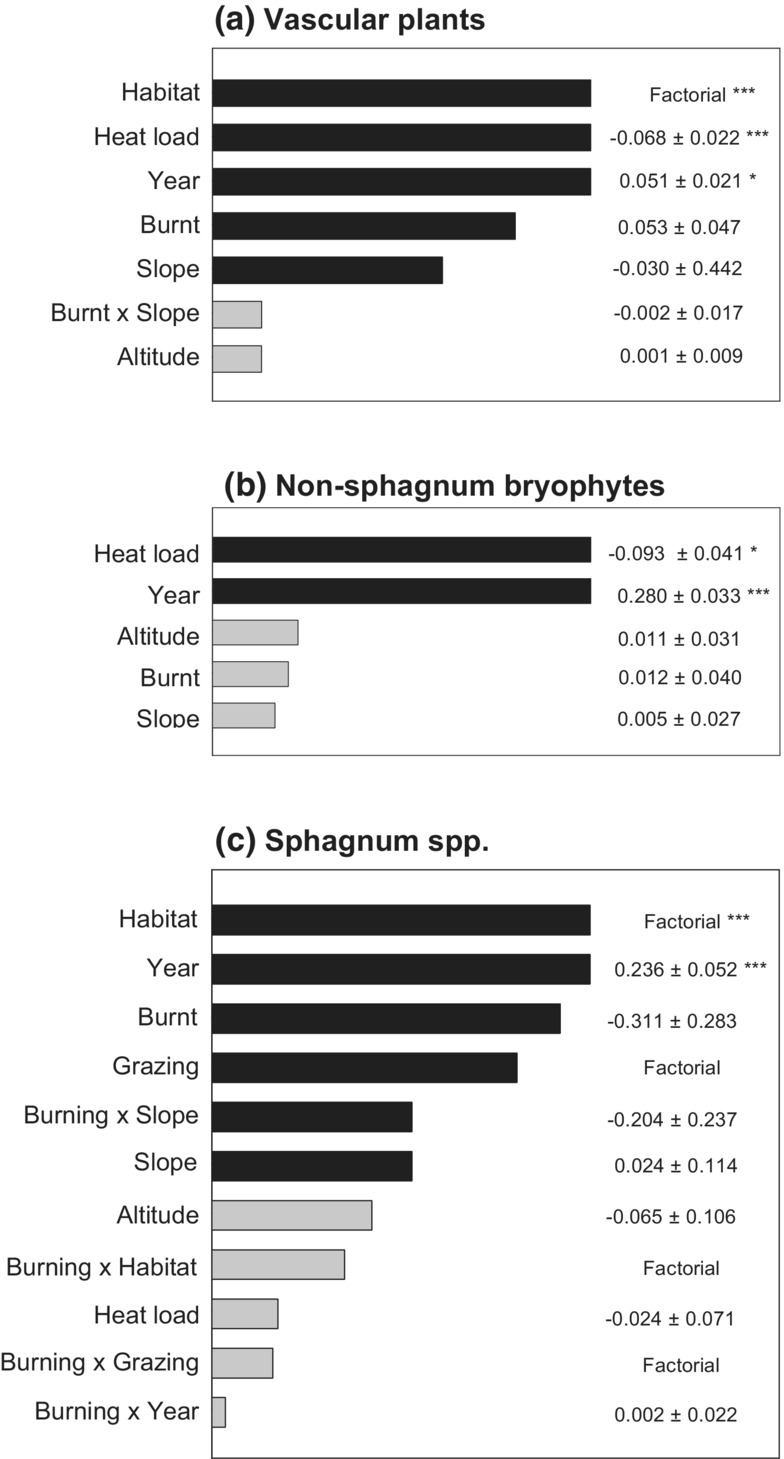
Relative importance of variables explaining variation in species richness of: (a) vascular plants, (b) non‐Sphagnum bryophytes, and (c) *Sphagnum* spp. Variables are ranked in order of the sum of their Akaike weights within the top set of models (i.e., models with ΔAIC <2). Black bars indicate variables included in the top model. Coefficients are averaged across the top set of models with zero value for models in which a variable was not included. Significance of individual model terms is indicated by * = *p* < .05, ** = *p* < .01, *** = *p <* .001.

Fifty‐nine vascular plant species were recorded, including eight shrub, four tree, eleven grass, eleven sedge, seven rush, one horsetail, fourteen herb, and three fern species. The median number of higher plant species per quadrat was 6 (min = 3, max =17). None of the species recorded were included in the Northern Ireland priority species list. Species richness of higher plants did not differ significantly between burnt and unburnt areas (*p* = .262). Species richness differed between Habitat types, being greater in dry and wet heath than in blanket bog (*p* < .001 in both cases). Dry and wet heath did not differ significantly from one another in terms of their species richness (*p =* .143). Vascular plant species richness was also negatively associated with Solar heat load (*p <* .001) and positively associated with Year (*p* = .013) (Figure 3).

**FIGURE 4:**
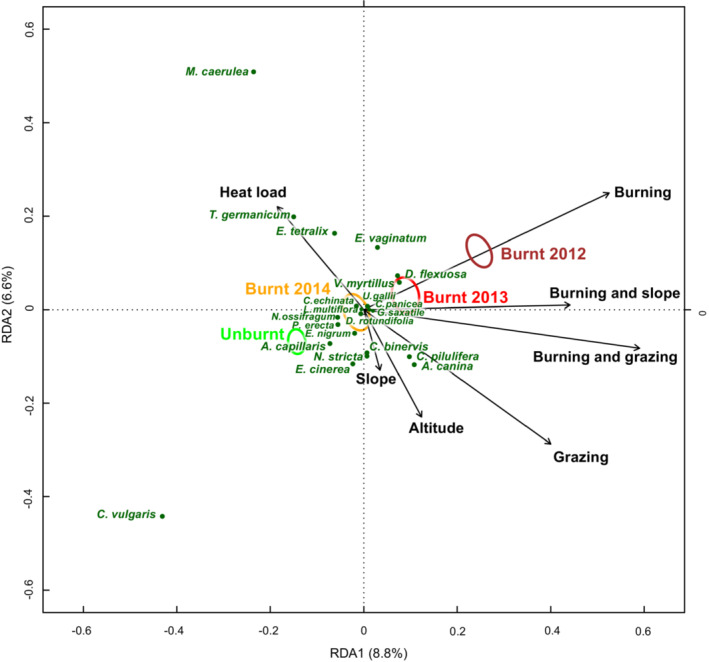
Relationship between environmental variables and the vascular plant community, after accounting for differences between sites by pRDA. Plant species are plotted where they were present in more than 10 quadrats and more than 5% of their variation was explained by the RDA model are plotted. Colored ellipses show standard error on the mean location of burnt quadrats in 2014 (dark red), 2013 (red), and 2012 (orange) and unburnt quadrats (green); illustrating the return of higher plant communities toward the baseline plant community in the 3 years after wildfires.

The species composition of the vascular plant community differed significantly between burnt and unburnt quadrats (Figure 4). These differences were influenced by the interaction between Burning and Grazing (*p* = .001), Burning and Habitat (*p* = .002), Burning and Slope (*p* = .015), and Burning and Year (*p =* .001). Altitude and Solar heat load also had a significant effect on plant community (*p* = .001 in both cases). Differences between sites explained 22.0% of the variation in the vascular plant community, and whilst all other environmental variables explained a further 18.6%.

Burnt areas had a lower abundance of ling heather (*Calluna vulgaris*), round‐leaved sundew (*Drosera rotundifolia*), crowberry (*Empretrum nigrum*), bog asphodel (*Narthecium ossifragum*), and deer grass (*Trichophorum germanicum*) which are all positive indicator species for blanket bog and wet heath in the UK (JNCC, 2009). However, burnt areas were associated with an increase in the abundance of common bilberry (*Vaccinium myrtillus*) which is a positive indicator species for blanket bog and wet heath and hare's tail cotton grass (*Eriophorum vaginatum*) which are both positive indicator species for blanket bog (JNCC, 2009). The abundance of the common grass species “wavy hair grass” (*Deschampsia flexuosa*) was also associated with burnt areas. Grazing was negatively associated with deer grass and cross‐leaved heath heather (*Erica tetralix*), a positive indicator species of blanket bog, wet heath and dry heath, and with hare's tail cotton grass (*Eriophorum vaginatum*) which is a positive indicator species for blanket bog (JNCC, 2009). Grazed areas had an increased abundance of graminoids including velvet bent grass (*Agrostis canina*) and matgrass (*Nardus stricta*), and sedges such as pill sedge (*Carex pilulifera*) and green‐ribbed sedge (*Carex binervis*). Areas which were both burnt and grazed had common graminoid species associated with both factors (e.g., wavy hair grass, velvet bent grass, matgrass, and pill sedge), but a lower abundance of the indicator species hare's tail cotton grass, than burnt areas which had lower levels of grazing pressure. Recovery of the vascular plant community was evident with plant communities moving along the vector for the effect of burning toward unburnt community composition (Figure 4).

### 3.3 Non‐*Sphagnum* bryophytes

**FIGURE 5:**
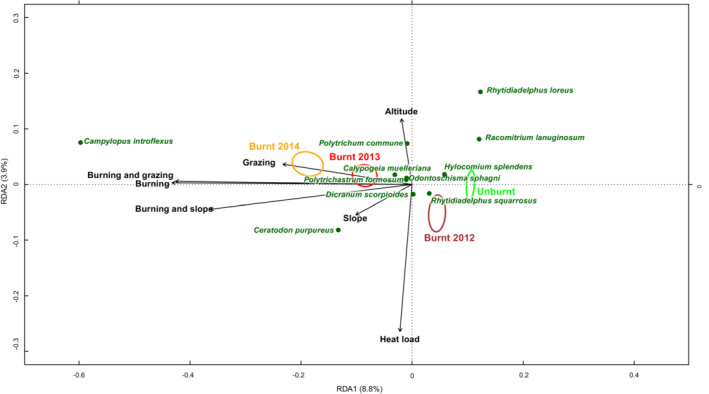
Partial Redundancy Analysis (pRDA) plot showing relationship between environmental variables and non‐sphagnum bryophyte cover after accounting for variation between sites by pRDA. Bryophyte species were plotted where they occurred in 10 or more quadrats in the study, and environmental variables explained more than 5% of the variation in their abundance. Colored ellipses show standard error on the mean location in the biplot space of burnt quadrats in 2014 (dark red), 2013 (red), and 2012 (orange) and unburnt quadrats (green); illustrating that burnt quadrats did not return toward the unburnt bryophyte community composition over the 3 years of the study.

Forty‐eight species of bryophytes other than *Sphagnum* spp. were recorded, including 31 species of moss and 17 species of liverwort. The median number of bryophyte genera per quadrat was 3 (min = 0, max = 10). Richness of non‐*Sphagnum* bryophyte species was not significantly associated with Burning but was negatively associated with Solar heat load (i.e., richness was higher in cooler areas) and increased between years (Figure 3). Burning was significantly associated with the community composition of non‐*Sphagnum* species (*p* = .001), indicating that although the overall number of species did not differ between burnt and unburnt areas the type of species did (Figure 5).

In addition to differences in community composition between burnt and unburnt areas, there was a significant interaction between Burning and Year (*p* = .001), Burning and Slope (*p =* .019), Burning and Habitat (*p =* .001), Burning and Grazing (*p* = .027), indicating that the response of species communities to burning depended on these other environmental variables (Figure 5). The interaction between Burning and Year was such that the community composition became more divergent over time and was characterized mainly by an increasing abundance of the alien pioneer acrocarp species *Campylopus introflexus* in burnt areas. Conversely, unburnt areas were characterized by pleurocarpous mosses including *Racomitrium lanuginosum, Rhytidiadelphus loreus,* and *Hylocomium splendens* which, although not peat‐forming, are considered indicators of “good condition” in blanket bog and heathland sites in the region (JNCC, 2009). Burning was also associated with a higher cover of the cosmopolitan pioneer acrocarp *Ceratodon purpureus*, and a marginal increase in *Polytrichiatrum formosum* and *Polytrichum commune*. Burning explained considerably less variation in the liverwort community. The three most common liverworts in both burnt and unburnt quadrats were *Calypogeia muelleriana, Odontoschisma sphagni,* and *Diplophyllum albicans*. Two liverwort species *Calypogeia muelleriana* and *Odontoschisma sphagni* were marginally more abundant in burnt areas (Figure 5). However, both were also found in unburnt quadrats are relatively common liverwort species in the upland flora. Altitude and Solar heat load also had a significant effect on community composition (*p* = .001 in both cases). Differences between sites explained 11.3% of the differences in the non‐*Sphagnum* bryophyte community, and the above environmental variables explained a further 17.0%.

### 3.4 Sphagnum spp.

Ten *Sphagnum* spp. were recorded. The median number of *Sphagnum* spp. per quadrat was 1 (min = 0, max = 5). The response of richness and diversity of *Sphagnum* spp. following burning was complex, with many factors contributing to the top set of models (Figure 3). Overall, *Sphagnum* spp. richness did not differ significantly between burnt and unburnt areas (*p* = .271). The most important factor associated with *Sphagnum* species richness was Habitat. As expected, *Sphagnum* species richness was significantly lower in dry heath habitats than in blanket bog (*p* < .001) or wet heath (*p* = .004) but did not differ significantly between wet heath and blanket bog (*p* = .418). In both wet heath and blanket bog, the median species richness was 1 and the most commonly occurring species was *Sphagnum capillifolium*. Species richness also differed between years (*p* < .001). Grazing, Slope and Burning and Slope alone were also included in the top model but were not significant when averaged across the top model set (*p* = .468, *p* = .391, and *p =* .837) (Figure 3).

**FIGURE 6:**
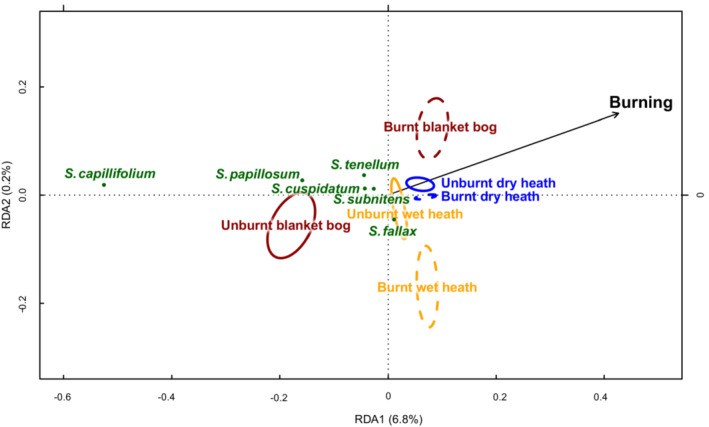
Partial Redundancy Analysis (pRDA) plot showing relationship between Sphagnum species composition and burning, after differences between sites are accounted for by pRDA. Species which were present in more than 10 quadrats are shown. Colored ellipses show standard error on the mean location in the biplot space of burnt and unburnt quadrats of each habitat type: blanket bog (dark red), dry heath (blue), and wet heath (yellow). The effect of “year” is not shown as there was no significant difference in communities in either burnt or unburnt quadrats between the 3 years of survey.

*Sphagnum* communities differed significantly between burnt and unburnt quadrats, and these differences depended on the habitat type (i.e., dry heath, wet heath, and blanket bog) (*p* = .001; Figure 6). The greatest differences were between unburnt and burnt quadrats on blanket bog habitats, where a higher diversity and abundance of *Sphagnum* spp. characterize the unburnt state of the habitat. Conversely, the smallest difference was seen between unburnt and burnt areas in dry heath, where *Sphagnum* spp. are uncommon (Figure 6). There was no significant effect of Year, or interaction of Burning and Year in the final model, indicating that there was no evidence of a change (e.g., recovery) of *Sphagnum* community composition in burnt areas over the 3 years of survey. Differences between Sites explained 26.4% of the variance in *Sphagnum* communities and a further 7.1% was explained by the interaction of Burning and Habitat.

Burning was negatively associated with the abundance of all frequently observed *Sphagnum* spp., namely *S. capillifolium, S. papillosum, S. tenellum, S. cuspidatum, S. subnitens,* and *S. fallax* (Figure 6). This negative association with burning was strongest for *S. capillifolium* and *S. papillosum,* which are key peat building species. Four other species were found in a small number of quadrats: *S. palustre*, *S. denticulatum, S. fuscum,* and *S. magellanicum* (present in nine, four, one and one quadrats, respectively). These showed no clear association either burnt or unburnt areas. *S. palustre* and *S. denticulatum* are relatively common species in the upland flora and showed *S. fuscum* and *S. magellanicum* are rare species of blanket bog habitats.

### 3.5 Ground beetles (Carabidae)

Thirty‐seven species of ground beetles were recorded including the Northern Ireland Priority Species *Carabus clatratus* (recorded at Cuilcagh). Species deemed “nationally scarce” in Great Britain were recorded at Cuilcagh and Slievebeagh (*Carabus nitens*) and Glennasheevar (*Cymindis vaporariorum*). A total of 3511 individuals were recorded, from 18 genera. The most commonly occurring genus was *Abax* (54%), with a single species *Abax parallelepipedus*. The second and third most common genera were Carabus (14%) (primarily *Carabus glabratus* and *Carabus problematicus*) and Pterostichus (14%) (primarily *Pterostichus niger, Pterostichus rhaeticus, Pterostichus madidus,* and *Pterostichus diligens*). Smaller proportions of the genera *Nebria* (<11%), *Notiophilus* (3%), *Cychrus* (1%), *Patrobus* (<1%), *Agonum* (<1%), *Leistus* (<1%), *Trechus* (<1%), *Bradycellus* (<1%), *Calathus* (<1%), *Loricera* (<1%), *Bembidion* (<1%), *Amara* (<1%), *Cymindis* (<1%), *Dyschirius* (<1%), and *Limodromus* (<1%) were observed. One percent of specimens could not be identified to species level.

**FIGURE 7:**
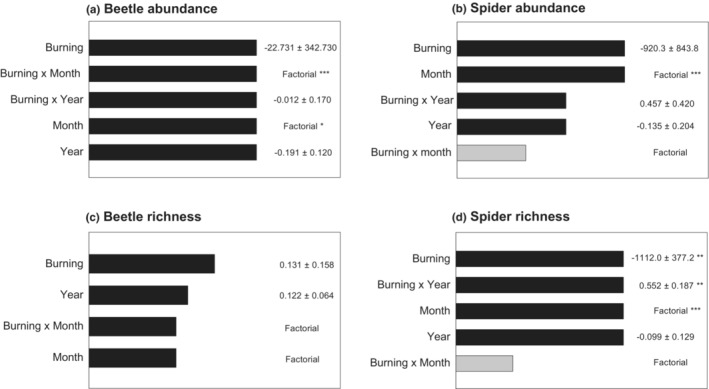
Relative importance of variables in explaining variation in (a) beetle abundance (b) beetle species richness, (c) spider abundance, and (d) spider species richness, based on model averaging. Variables are ranked in order of the sum of their Akaike weights within the top set of models (i.e., models with ΔAIC <2). Coefficients are averaged across the top set of models with zero value for models in which a variable was not included. Black bars indicate variables included in the top model. Significance of individual model terms is indicated by * = *p* < .05, ** = *p* < .01, *** = *p <* .001.

The difference in the abundance of ground beetles between unburnt and burnt areas was influenced by Month (*p <* .001; Figure 7). In the summer months (June, July, and August), ground beetle abundance was higher in unburnt areas than in burnt areas (mean abundance per trap in unburnt/burnt traps = 10.0/7.5, 10.8/8.4, 11.9/7.6 in June, July, and August, respectively). However, at the end of the sampling season (September), ground beetle abundances were higher in burnt than unburnt areas, (mean abundance per trap in unburnt/burnt traps = 6.4/8.9). These differences most likely relate to changes in the relative utilization of burnt areas by ground beetles later in the season, rather than an overall change in ground beetle abundance. Abundance also differed significantly between months irrespective of burning, such that beetle abundances were highest July and lowest in September (*p* = .033). Beetle species richness did not differ significantly between burnt and unburnt areas, or between years and months (Figure 7).

**FIGURE 8:**
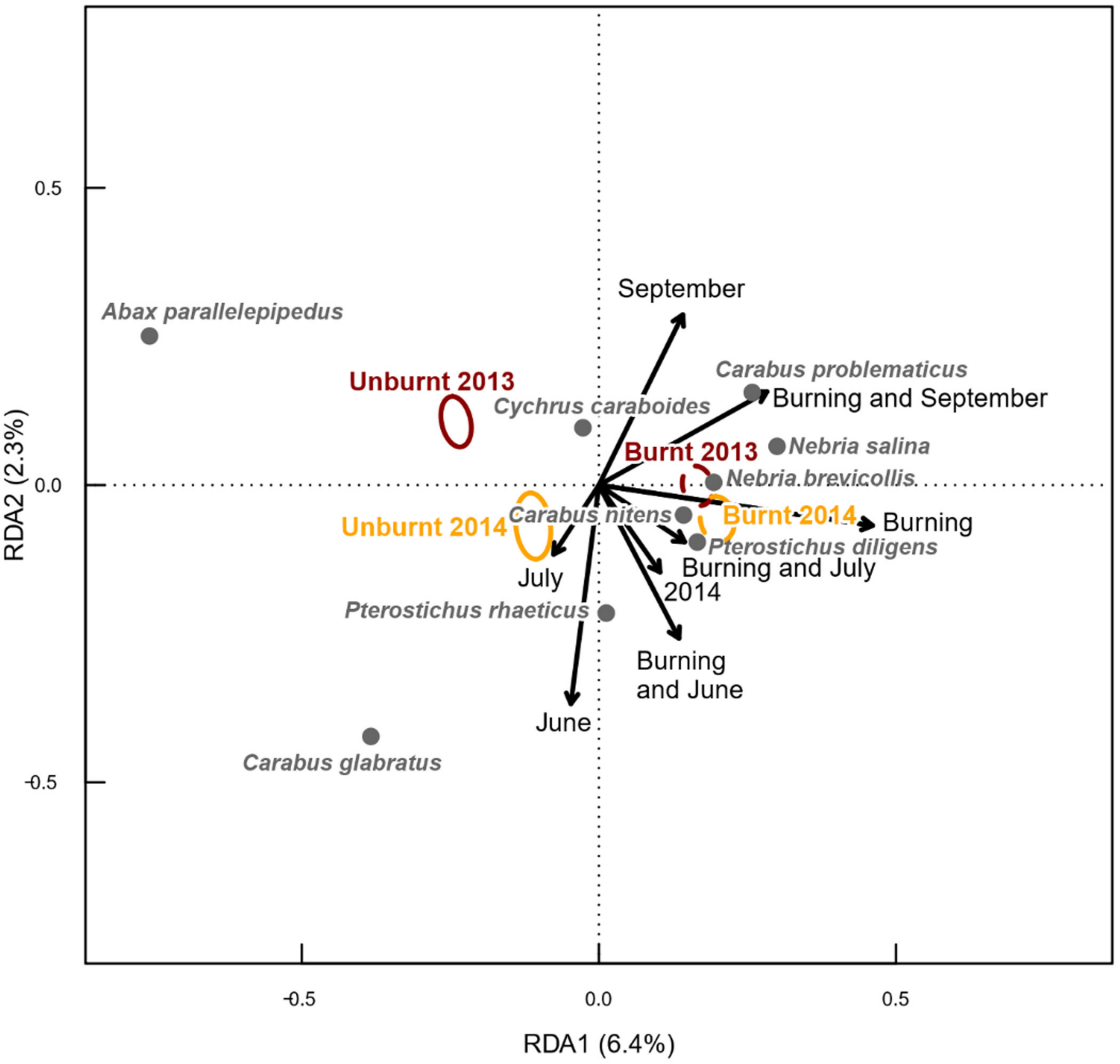
Partial Redundancy Analysis (pRDA) plot showing relationship between ground beetle species composition and burning, after accounting for differences between sites by pRDA. Species are plotted if they were recorded at least 30 times and more than 5% of the variance in their abundance was explained by the fitted RDA model, after accounting for differences between sites. Colored ellipses show standard error on the mean location in the biplot space of burnt and unburnt samples in 2013 (dark red) and 2014 (yellow), respectively. Dashed circles indicate burnt samples and solid lines indicate unburnt locations.

Ground beetle community composition (Figure 8) differed significantly between burnt and unburnt areas, and there was a significant interaction between Burning and Month (*p* = .009) and Burning and Year (*p* = .020). This interaction between Burning and Year marginally increased the divergence of ground beetle species communities between burnt and unburnt areas in 2014 relative to 2013. Differences between sites accounted for 14.1% of the variance in ground beetle communities, and a further 11.0% was accounted for by the combination of Burning, Month, and Year. Ground beetle species which were associated with unburnt areas included the widespread common upland species *Abax parallelepipedus, Cychrus carabidoides,* and *Agonum fuliginosum* and the common but more localized peat species *Carabus glabratus*. Burnt areas were associated with higher abundances of the widespread and common upland species *Carabus problematicus, Nebria salina*, *Nebria brevicollis,* and *Pterostichus diligens*. Burnt areas were also associated with the much rarer species *Carabus nitens* (considered “Nationally scarce” in Great Britain) (Figure 8). No conclusions could be reached about the effect of burning on the priority species *Carabus clatratus* due to small sample size but it was recorded in both unburnt and burnt patches within the single site where it occurred. The nationally scarce species *Cymindis vaporariorum* cannot be analyzed for the same reason, as this species was recorded in only one trap in an unburnt area of a single site.

### 3.6 Spiders (Araneae)

Seventy‐five species of spiders were recorded across the five ASSI's included in the invertebrate analyses. A total of 1063 individuals were caught. The most commonly occurring family was Lycosidae (51%), primarily *Pirata uliginosus, Trochosa terricola,* and *Pardosa pullata*. The second most common family was Linyphiidae (37%), which was represented by a broader range of species the most abundant of which were *Antistea elegans, Gonatium rubens, Walckenaeria acuminata, Agyneta olivacea, Lepthyphantes zimmermanni, Centromerita concinna, Saaristoa abnormis, Silometopus elegans, Micrargus herbigradus, Hilaira pervicax,* and Walckenaeria *cuspidata*. Small proportions of Clubionidae (<1%), Gnaphosidae (<1%), Hahniidae (3%), Liocranidae (1%), Mimetidae (<1%), Tetragnathidae (<1%), Theridiidae (3%), and Thomisidae (1%) were observed. Eleven specimens could not be identified to family level.

There was no significant difference between spider abundance between burnt and unburnt areas or between years (Figure 7; *p* = .277 and *p =* .510 respectively). Spider abundance differed significantly between months being lower in September than June (Figure 7; *p <* .001). Species richness of spiders was significantly higher in unburnt areas, with a significant interaction between burning and year (Figure 7; *p* = .003). Spider species richness in burnt areas was higher in 2014 than in 2013, potentially indicating some recovery. Species richness of spider communities also differed between the 2 months surveyed and was significantly lower in September than in June (*p* < .001).

**FIGURE 9:**
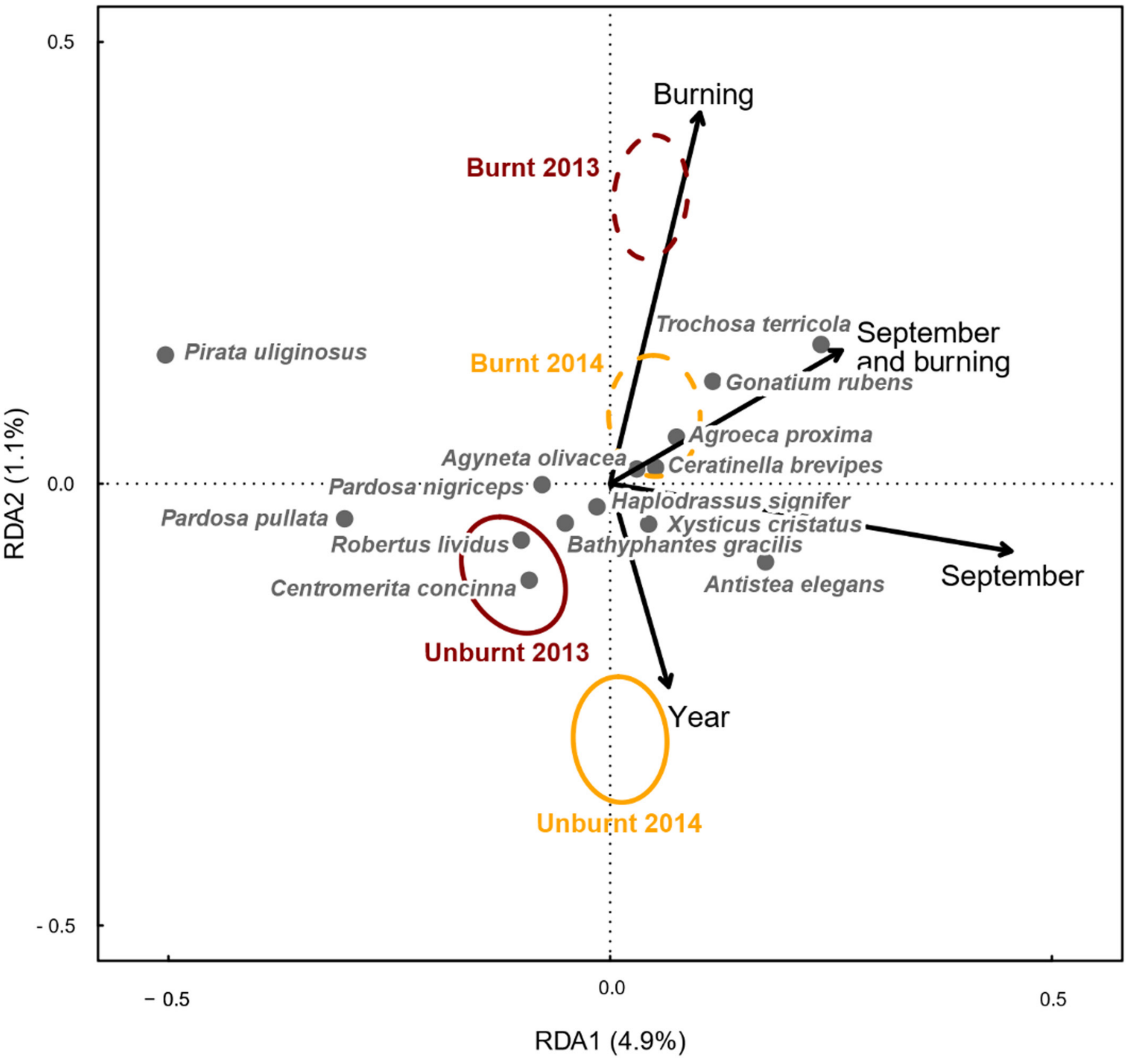
Partial Redundancy Analysis (pRDA) plot showing relationship between spider species composition and burning, after accounting for differences between sites by pRDA. Species are plotted if they were recorded at least five times and more than 5% of the variance in their abundance was explained by the fitted RDA model, after accounting for differences between sites. Colored ellipses show standard error on the mean location in the biplot space of burnt and unburnt samples in 2013 (dark red) and 2014 (yellow), respectively. Dashed circles indicate burnt samples and solid lines indicate unburnt locations.

There was a significant association between burnt areas and spider community composition, and this association differed between months (Figure 9; *p* = .017). Spider communities also differed between survey years (*p =* .008), but there was no interaction between Burning and Year (i.e., no evidence of recovery or deterioration of community composition in burnt areas between the 2013 and 2014). Differences between sites explained 8.9% of the variation in spider communities, and a further 7.0% was explained by the combination of the Burning, Month, and Year (Figure 9). Unburnt communities were characterized by a higher abundance of some wolf spider (Lycosidae) species, namely *Pirata uliginosus* and *Trochosa spinipalpis* (in June) and *Trochosa terricola* (in September). *Pirata uliginosus* and *Trochosa spinipalpis* have been previously noted to be a good peat bog indicator species in western Britain whilst *Trochosa terricola* which is common in peat bogs but also tolerates drier habitats (Scott et al., 2006). In September, unburnt sites were also characterized by a higher abundance of the *Gonatium rubens* (Linyphiidae) and *Agroeca proxima* (Liocranidae) which are common in the UK and found in a wide range of habitat types. Conversely, burnt areas were characterized by higher abundances of *Robertus lividus* (Theridiidae) and *Centromerita concinna* (Linyphiidae) which are considered appropriate for peatbogs in western Britain (Scott et al., 2006), but are also widespread and common in other habitat types. In September, burnt areas were characterized by a higher abundance of the *Antistea elegans* (Hahniidae) which is also considered to be a good indicator for peat bogs in western Britain by Scott et al. (2006) and by the common species *Xysticus cristatus* (Thomisidae) and is found in a wide range of habitat types.

There was no strong association between spider families and burning, with the two most abundant families: wolf spiders (Lycosidae) and money spiders (Linyphiidae) occurring in both burnt and unburnt areas. Rather, associations with burnt or unburnt habitat types were found to be species‐specific, for example, within the genus *Pardosa (*Lycosidae)*, P. uliginosus* was found to be strongly associated with unburnt areas, whilst the abundant species *Pardosa pullata* was found equally frequently in both burnt and unburnt areas.

## 4 DISCUSSION

Our findings suggest that across all taxa differences in wildfire areas were mainly characterized by changes in species‐level community composition. Such changes were evident across all species groups examined: vascular plants, bryophytes, ground beetles, and spiders. Furthermore, species richness did not differ significantly between burnt and unburnt areas for vascular plants, bryophytes, or ground beetles. With the exception of *Sphagnum* spp., all broad taxonomic groups had some species with higher abundance in burnt areas indicating that some species may take advantage of novel conditions postwildfires to increase population abundance. In addition, the regrowth of shrub cover and thus overall structure were relatively quick at all sites apart from Slieve Beagh where prefire shrub cover was least (Figure 2).

### 4.1 Vascular and non‐vascular plant communities

Postwildfire plant community composition shifted away from species characteristic of these protected upland habitats, toward generalists and rapidly colonizing species, allowing the establishment of an invasive species of bryophyte. Indicator species of blanket bog, wet heath, or dry heath such as ling heather, crowberry, deergrass and bog asphodel, and bryophytes including *Sphagnum*, *Rhytidiadelphus,* and *Racomitrium* species, declined in burnt areas. However, two commonly used indicator species, common bilberry and hare's tail cotton grass, did not show this pattern and were positively associated with burnt areas. Root and leaves of the latter species have comparable growth rates ensuring good recovery after fires (Kummerow et al., 1988). Concurrently, generalist species, such as purple moor grass, increased in burnt areas as did pioneer acrocarp mosses including *Ceratodon purpureus* and *Campylopus introflexus*.

Burning was negatively associated with the abundance of all frequently observed *Sphagnum* spp. This negative association with burning was strongest for *S. capillifolium* and *S. papillosum,* which were common in unburnt areas and are key peat‐forming species. The peat‐forming species *S. fuscum* and *S. magellanicum* had no association with burnt or unburnt areas and were also rare on blanket bog in this study. Results from studies of prescribed burning suggest that hummock‐forming species such as *S. capillifolium* may survive fires better due to their increased water retention (Peatscapes *Sphagna as management indicators of research,* 2008). The reduction of *Sphagnum* spp. evident here may have resulted from the higher heat intensity or longer duration of uncontrolled wildfires. Previous authors have noted that the prescribed burning of patches of upland may increase the overall species diversity of the site, by creating more open areas in heather‐dominated landscapes (e.g., Davies et al., 2010; Harris et al., 2011). However, we found that following wildfires on sites of conservation interest with relatively high plant diversity, the species composition shifts toward common, pioneer species and away from characteristic upland species. Wildfires similarly may affect communities in “wet hollows” more than those forming a dense “lawn” due to the latter's better water retention (Blier‐Langdeau et al., 2022).

Research on the impact of prescribed burning on areas of upland heath suggests that the recovery of a *Calluna‐*dominated community occurs in upland heath habitats over a period of 20–25 years. However, many of these studies have been conducted following prescribed burning on previously degraded or intensively managed sites. It is unclear to what extent research on prescribed burning can be generalized to large uncontrolled wildfires on more botanically diverse sites (Worrall et al., 2010). Present results include some similarities with previous studies, such as the relatively rapid recovery of heather species (*C. vulgaris* and *E. tetralix*) and the increase in graminoid species postwildfire (e.g., *D. flexuosa*). However, we also found some differences from previous studies, for example, the negative impacts on key indicator species such as bog asphodel (*N. ossifragum*) and round‐leaved sundew (*D. rotundifolia*) which are characteristic of wetter sites*,* and which, to our knowledge, were not present in previous studies of moorland burning in the UK. The absence of a focus on these species in previous studies may relate to shifting baselines, where species composition postdisturbance is being compared with an already reduced or altered species community.

### 4.2 *Campylopus introflexus*: An invasive alien bryophyte

Grazing increased the frequency of *C. introflexus*. The main change in the bryophyte community composition of burnt areas over the period of this study was also an increase in *C. introflexus* and further divergence from community composition in unburnt areas was primarily characterized by pleurocarpous mosses. *Campylopus introflexus* is an alien species to Europe from the southern hemisphere first introduced to the UK in 1941 (Richards, 1963). Previous authors have suggested that it can outcompete other moss species and lichens following disturbance. However, as much of the previous research has been conducted in dune and alkaline grassland systems (Klinck, 2010), further research is required on the implications of this species for upland habitats. Research conducted on the competitive interactions of *C. introflexus* with ling heather is equivocal, suggesting that *C. introflexus* reduces germination of ling from the seed bank but increases growth rates once seedlings are established (Equihua & Usher, 1993).

### 4.3 Ground beetle and spider communities

Changes in the species composition of arthropods included increases in the abundance of ground beetle species *Carabus nitens* which is classified as “nationally scarce” in Great Britain in burnt areas. However, this species may be increasing across the UK perhaps due to changes in climate or upland management (Brooks et al., 2012). Thus, it is unclear whether *C. nitens* will be a UK conservation concern in the future. The spider *Antistea elegans* was also found in higher abundances in burnt areas despite being considered characteristic of peat bogs in good condition (Scott et al., 2006). These changes may reflect higher temperatures in the sparser vegetation cover and exposure to solar radiation in burnt heathland. Changes in higher trophic levels involving carnivorous arthropods may initiate a trophic cascade influencing all parts of the upland trophic web. Driessen and Kirkpatrick (2017) worked on the impact of mostly planned fires on invertebrate taxa in Tasmanian moorland using a “space‐for‐time” approach. This suggests that vegetation density and soil productivity affect rate of change in invertebrate assemblages after fire but ultimately ground and foliage invertebrate fauna return to the prefire state in this fire‐prone ecosystem. This may be less likely where fire is not a normal occurrence. Prescribed burning of cool temperate moorland is associated with complex successional pathways in beetle and spider community composition, and is related to the recovery of vegetation structure. Thus, planned burning may produce a mosaic of burnt and unburnt patches of heather of different ages and structure which enhances overall diversity and sustains rarer species of beetles and spiders (Eyre et al., 2003; McFerran et al., 1995). Wildfires may not have such predictable outcomes if they affect large, continuous areas of peatland, limiting dispersal and population recovery, and burn more deeply, increasing direct mortality of invertebrates as well as removing the vegetation and surface heterogeneity required to shelter beetles and spiders from predators or survive overwinter. However, overall vegetation structure was determined by shrub cover at five of the six sites surveyed and this started recovery quickly after fire suggesting that structure which is key for spiders and beetles had commenced within the timeframe of the study. However, as noted above, differences remained or were increasing within the bryophyte flora at the soil surface.

### 4.4 Conclusion and implications for management

Present results were largely consistent with our initial hypotheses: recovery of plant and arthropod communities were incomplete in the short term; heathland and bogland specialist plants, particularly *Sphagnum* spp., were more affected by wildfires than generalist plants; and community composition of spiders and carabids, that is, abundance of particular species was more affected than overall species richness. This study was conducted as part of a wider study on the aftermath of wildfires in uplands designated for their ecological interest, which also found changes in soil chemistry (Kelly et al., 2018), reduced plant species diversity in the seed bank (Kelly et al., 2016), and greater impacts of wildfire on specialist upland and peatland birds compared with more generalist bird species (Reid et al., 2023) (Table 2). Recolonization by plants postfire is dependent on a number of factors including resprouting (from roots or stems) and seed banks and landscape scale factors such as dispersal (Kelly et al., 2016; Shepherd et al., 2021). Postfire recolonization from nearby peatland and nonpeatland locations may be key in determining postfire species composition (Shepherd et al., 2021). Longer term research is required to establish whether the initial shift in the plant community observed in the present short‐term study, is indicative of a longer term shift in vegetation composition. Nonetheless, the short‐term increase in the prevalence of graminoid species, reduction in *Sphagnum* moss, and associated drier vegetation has the potential to increase the risk of more frequent fire regimes (Kettridge et al., 2015), alter nutrient cycles (Gogo et al., 2011; Kelly et al., 2018), increase soil loss (Morán‐Ordóñez et al., 2020), and reduce carbon sequestration (Lin et al., 2021).

**TABLE 2:** Observed changes in ecological characteristics within three and a half years, between designated moorland burnt by wildfires and comparable unburnt areas

Key differences in designated areas burnt by wildfires and not burnt by wildfires >increase <decrease	Evidence of convergence in characteristics of burnt and unburnt areas
>Soil phosphorus (mg/l) and Calcium (mg/l)	NO
>Bare peat surface	YES
Changes in proportion of broad plant groups (e.g., shrubs, herbs, graminoids, and bryophytes)	YES
<Key peatland indicator species 1. Vascular plants Ling heather (*Calluna vulgaris*), bog asphodel (*Narthecium ossifragum*), round‐leaved sundew (*Drosera rotundifolia*), crowberry (*Empetrum nigrum*) and deer grass (*Trichophorum germanicum*)	1. YES
2. Non‐sphagnum bryophytes *Racomitrium lanuginosum,* *Rhytidiadelphus loreus and* *Hylocomium splendens*	2. NO
3. Sphagnum	3. NO
4. Spiders (Scott et al., 2006) *Pirata uliginosus* and *Trochosa spinipalpis*	4. NO
5. Breeding birds (Neil et al. in press) Redshank (*Tringa totanus*)*,* Meadow pipit (*Anthus pratensis*), Stonechat (*Saxicola rubicola*), raptors	5. NO
<Key peat‐forming *Sphagnum* species	NO
>Alien moss species *Campylopus introflexus*	NO
<Spider species richness	YES
<Bird species richness	YES

*Note*: Based on Kelly et al. (2016, 2018) and Reid et al., 2023.

Given the potential for detrimental impacts on carbon sequestration, water, and soil retention and nutrient cycling and changes in species composition evident immediately after anthropogenic wildfires, a precautionary approach is advisable to prevent further wildfires and protect these key upland habitats. For example, cutting may be a viable alternative to prescribed burning on heather moorland (Sanderson et al., 2020) reducing the chance of collateral wildfires. Recurring, severe wildfires will inevitably lead to fragmented isolated peatland ecosystems on the edge of their climatic envelopes with diverse animal and plant species facing extinction (Kettridge et al., 2019). Shallow carbon‐rich soils, such as prevail in blanket peatland, are particularly vulnerable to the impacts of wildfires (Wilkinson et al., 2020). The latter authors suggest that burn severity is inversely related to peat depth up to 0.66 m and regulated by the water table. Maintaining water tables at their highest possible levels by blocking drains, preventing peatlands from drying out during periods of drought, should be prioritized in heathland management. Education of landowners, land managers and the public, vigilance and management of access to vulnerable moorland during drought conditions, should reduce the likelihood of prescribed burns getting out of control, and accidental wildfires.

## AUTHOR CONTRIBUTIONS

**Ruth Kelly:** Data curation (lead); formal analysis (lead); investigation (lead); methodology (lead); writing – original draft (lead); writing – review and editing (equal). **Ian W Montgomery:** Conceptualization (equal); funding acquisition (equal); methodology (equal); project administration (equal); resources (equal); supervision (equal); writing – original draft (equal); writing – review and editing (equal). **Neil Reid:** Conceptualization (equal); data curation (equal); formal analysis (equal); funding acquisition (equal); methodology (equal); project administration (equal); resources (equal); supervision (equal); writing – review and editing (equal).

## CONFLICT OF INTEREST

We declare no competing interests.

## Data Availability

All data are available on Dryad at: https://doi.org/10.5061/dryad.fbg79cnzz.
